# EASIX, a new tool to predict response and refractoriness in immune-mediated thrombotic thrombocytopenic purpura

**DOI:** 10.3389/fimmu.2025.1700907

**Published:** 2025-11-17

**Authors:** Silvia Escribano-Serrat, María Queralt Salas, Cristina Pascual-Izquierdo, Manuel Fernández Villalobos, Inés Gómez-Seguí, Teresa Fidalgo, Marta Fernández Docampo, Jorge Martínez Nieto, Óscar Lamas, Miquel Lozano, Joan Cid, Paola Charry, Marc Pino, Patricia Molina, Blanca De Moner, Álex Ramos, Ana Belén Moreno-Castaño, Julia Martinez-Sanchez, Ginés Escolar, Maribel Díaz-Ricart

**Affiliations:** 1 Laboratory of Hemostasis and Erythropathology, Hematopathology, Pathology Department, CDB, Hospital Clínic de Barcelona, Barcelona, Spain; 2 Instituto de Investigaciones Biomédicas August Pi i Sunyer (IDIBAPS), Barcelona, Spain; 3 Hematopoietic Transplantation Unit, Hospital Clínic de Barcelona, ICAMS, Barcelona, Spain; 4 Hematology Department, Hospital Universitario Gregorio Marañón, Instituto de Investigación Gregorio Marañón, Madrid, Spain; 5 Hematology Department, Hospital Universitario La Fe, Valencia, Spain; 6 Centro Hospitalar Universitário de Coimbra, Coimbra, Portugal; 7 Área de Gestión Integrada de A Coruña, Instituto de investigación Biomédica de A Coruña (INIBIC), A Coruña, Spain; 8 Hospital Clínico San Carlos, Madrid, Spain; 9 Apheresis and Cellular Therapy Unit, Department of Hemotherapy and Hemostasis, ICAMS, Hospital Clínic de Barcelona, Barcelona, Spain; 10 Josep Carreras Leukaemia Research Institute, Hospital Clínic de Barcelona, Barcelona, Spain

**Keywords:** ITTP, EASIX, ADAMTS13 activity, biomarker, endothelial dysfunction

## Abstract

**Introduction:**

Immune-mediated thrombotic thrombocytopenic purpura (iTTP) is a life-threatening thrombotic microangiopathy resulting from severe ADAMTS13 deficiency. Caplacizumab accelerates platelet recovery, but ~15% of patients remain refractory, and endothelial/microvascular injury or low ADAMTS13 activity may persist despite remission, highlighting the need for biomarkers. We evaluated the Endothelial Activation and Stress Index (EASIX), an endothelial dysfunction surrogate, dynamics and ability to predict refractoriness and mortality in iTTP.

**Methods:**

Fifty-five adults receiving ≥2 therapies (corticosteroids, plasma exchange, rituximab, and/or caplacizumab) were studied. Clinical and laboratory data were collected at baseline, days 1–2, 7, 14, 21, 28, 35, and at treatment discontinuation, including clinical or ADAMTS13 relapses. EASIX was calculated at each time point; logistic regression and ROC analyses evaluated its predictive performance for refractoriness and mortality.

**Results:**

Median age was 47 years; 13% were refractory, and 7% died. In responders, EASIX dropped below 1 by day 7, earlier than ADAMTS13 recovery (day 21). Clinical relapses showed EASIX spikes (median 13.2), unlike ADAMTS13-only relapses. Baseline EASIX was higher in refractory patients (752 vs. 91; p=0.007), remaining elevated at days 7 and 14. Higher pre-treatment EASIX predicted refractoriness (OR = 1.003; p=0.021; AUC = 0.811; sensitivity 100%; specificity 58.7%) and mortality (OR = 1.004; p=0.027).

**Discussion:**

EASIX may help predict refractoriness and death, improving monitoring in iTTP.

## Introduction

Thrombotic microangiopathy (TMA) is a distinct pathological entity characterized by widespread endothelial injury, leading to the formation of platelet-rich thrombi that occlude the microvascular lumen, and result in ischemia, thrombocytopenia, and microangiopathic hemolytic anemia (1, 2). Thrombotic thrombocytopenic purpura (TTP), a rare but potentially fatal TMA, has an estimated incidence of 1.5–6 cases per million adults per year and is defined by severe deficiency of ADAMTS13 (<10%), either congenital or immune-mediated (iTTP) (1–4). This deficiency leads to the accumulation of ultra-large von Willebrand factor (vWF) multimers and uncontrolled platelet aggregation, causing widespread microvascular thrombi (1, 4).

Without treatment, TTP has a mortality rate exceeding 90%. Current therapeutic strategies have reduced mortality to below 10% in specialized centers, although 10-15% of patients remain refractory to initial therapies (4, 5). Despite these advances, early recognition and prompt initiation of treatment remain critical (2, 4, 5). First-line treatment for iTTP consists of daily therapeutic plasma exchange (TPE), corticosteroids (CS) and rituximab (2, 5–7). More recently, the addition of caplacizumab, an anti-vWF antibody, has significantly improved clinical outcomes by accelerating platelet recovery and reducing the risk of disease exacerbation (2, 5, 8–12). Platelet count and lactate dehydrogenase (LDH) remain essential biomarkers for assessing disease activity and treatment response. However, the rapid normalization of platelet counts with caplacizumab highlights the need for dynamic and individualized monitoring strategies, particularly in the early stages of therapy (4, 5, 13).

The Endothelial Activation and Stress Index (EASIX), initially developed in the setting of allogeneic stem cell transplantation to predict treatment response and survivorship in patients with graft-versus-host disease, has emerged as a surrogate marker of endothelial dysfunction and a predictor of clinical outcomes and vascular complications, including transplant-associated TMA and sinusoidal obstruction syndrome. Its utility has subsequently been validated across diverse clinical contexts, such as hematologic malignancies, CAR-T cell therapy, sepsis, and COVID-19 (14–27).

Given the central role of endothelial damage in iTTP, this study aimed to determine the dynamic behavior of EASIX in patients with iTTP and explore its potential as a predictive marker of refractoriness and mortality.

## Methods

### Patient selection

This was an international, multicenter retrospective cohort study involving 6 institutions from Spain and Portugal (Hospital Clínic de Barcelona, Hospital Universitario Gregorio Marañón, Hospital Universitario de la Fe, Centro Hospitalar e Universitário de Coimbra, Complejo Hospitalario Universitario de A Coruña, and Hospital Clínico San Carlos). All patients ≥18 years olddiagnosed with iTTP between 2015 and 2024, defined by ADAMTS13 activity <10% with detectable anti-ADAMTS13 antibodies, who received at least two of the following treatments at onset: CS, TPE, rituximab, or caplacizumab, were included.

This study was approved by the Ethics Committee of Hospital Gregorio Marañón (COAG-PTT-2021-01) and Hospital Clinic de Barcelona (HCB/2022/0073), and was conducted in accordance with the principles outlined in the Declaration of Helsinki.

### Clinical and laboratory data

Demographic and clinical data were collected retrospectively and updated in May 2025, including patients’ vital status (alive or deceased). Laboratory variables were collected at the following predefined time points: at onset (day 0), during debut follow-up (on days 1-2, 7, 14, 21, 28, and 35 after the start of targeted treatment) and on the day of discontinuation of the last targeted therapy agent. If clinical or ADAMTS13 relapse, or isolated ADAMTS13 activity decline occurred, laboratory data were also collected on the first day of the event.

Complete blood counts were assessed using automated analyzers in each center. ADAMTS13 activity was measured using fluorescence resonance energy transfer (FRETS) assay or ELISA methods, depending on the center. Following internal validation of the FRETS assay, determinations were performed in parallel in the absence and presence of a bilirubin inhibitor (bilirubin oxidase, Sigma-Aldrich, Spain), to prevent potential interference from bilirubin. Anti-ADAMTS13 antibodies were assessed using ELISA methods.

EASIX was calculated using the following formula: creatinine (mg/dL)*LDH (U/L)/platelets (x10_9_/L). If clinical or ADAMTS13 relapses, or an isolated ADAMTS13 activity decline occurred, EASIX was also calculated on the first day of the event.

### Main definitions

Targeted treatment was defined as the administration of TPE, rituximab, and/or caplacizumab. Clinical and ADAMTS13 remission, exacerbations, and clinical and ADAMTS13 relapses were defined according to the criteria established by *Cuker* et al. (13, 28). Isolated ADAMTS13 decline refers to a single drop in ADAMTS13 activity below 20%, not confirmed on subsequent testing and not associated with any clinical signs or symptoms. Refractoriness, as defined by *Scully* et al., was characterized by persistent thrombocytopenia (< 50×10^9^/L) and persistently elevated LDH despite at least five sessions of TPE plus CE, with or without rituximab (28). For those receiving caplacizumab, refractoriness was defined by any of the following: 1) death attributable to iTTP, 2) failure to achieve clinical remission within five days, characterized by a platelet count ≥150x10^9^/L along with normalization of LDH levels or resolution of organ damage, 3) emergence of new signs of organ injury despite ongoing treatment.

### Statistical analysis

Descriptive statistics were reported as medians with interquartile ranges (IQR) for continuous variables, and as counts with percentages for categorical variables. Wilcoxon rank sum test was used to evaluate the association between continuous variables.

To assess the correlation between EASIX values and clinical outcomes, a logistic regression analysis was conducted, including EASIX as a continuous independent variable. Results are reported as odds ratios (OR) with 95% confidence intervals (CI) and corresponding p-values.

To further explore the predictive accuracy of EASIX at day 0 and day 1, receiver operating characteristic (ROC) curve analyses were performed. Additionally, to determine whether it provides prognostic information beyond its individual components, separate analyses were conducted using platelet count and LDH levels at days 0 and 1.

For mortality prediction analyses, only deaths directly attributable to iTTP were considered, in order to ensure disease-specific prognostic evaluation.

All P-values were two-tailed, and p-values <0.05 were considered statistically significant. Statistical analyses were performed using R version 4.3.2 (R Foundation for Statistical Computing, Vienna, Austria, https://www.R-project.org/) with the “gtsummary” and “pROC” packages.

## Results

### Clinical data

A total of 55 patients were included. Baseline characteristics are detailed in **Table 1**. The median age at iTTP onset was 47 years (range: 21–77), and 34 (62%) patients were females. At diagnosis, all patients presented with thrombocytopenia and 53 (96%) with anemia. The most common clinical manifestations were neurologic (56%) and gastrointestinal (23%) symptoms.

**Table 1:** Clinical data.

Variable	Overall (n=55)^1^
Age (years)	47 (36, 61)
Sex (female)	34 (62%)
Signs and symptoms at debut	
Thrombocytopenia	55 (100%)
Anemia	53 (96%)
Neurological	31 (56%)
Gastrointestinal	13 (23%)
Renal	3 (5%)
Cardiac	2 (3%)
Treatment at debut	
Corticosteroids	54 (98%)
Median days	56 (35, 76)
Therapeutic plasma exchange	53 (96%)
Median days	7 (4,11)
Rituximab	43 (78%)
Median days	21 (21, 22)
Caplacizumab	41 (75%)
Median days	31 (22, 37)
Treatment combinations	
CS + TPE + rituximab + caplacizumab	33 (60%)
CS + TPE + rituximab	9 (16%)
CS + TPE + caplacizumab	6 (10%)
CS + TPE	6 (10%)
Other	1 (1%)
Complications at debut	19 (35%)
Infectious	8 (14%)
Cardiological	6 (10%)
Bleeding	4 (7%)
Pulmonary	1 (1%)
Refractory at debut	7 (13%)
Exacerbations at debut	5 (9%)
Clinical relapses	13 (24%)
ADAMTS13 relapses	6 (11%)
Isolated ADAMTS13 declines	9 (16%)
Death	4 (7%)

^1^Median (IQR) or Frequency (%)

CS, corticosteroids; TPE, therapeutic plasma exchange.

Regarding treatment, 54 patients (98%) received CS, 53 (96%) TPE, 43 (78%) rituximab, and 41 (75%) caplacizumab. The combination of CS, TPE, rituximab, and caplacizumab was the most frequently administered regimen (n=33, 60%). One patient did not receive CSdue to a prior history of CS-induced psychosis. Notably, two patients did not receive TPE as they were treated within the context of a clinical trial where TPE was not included in the treatment protocol.

Complications at debut were observed in 19 patients (35%), predominantly of infectious origin (14%). Refractoriness occurred in 7 patients (13%), of whom five had received caplacizumab. Clinical relapse was documented in 13 patients (24%) and ADAMTS13 relapse in 6 patients (11%). Four (7%) patients died.

### Laboratory data and EASIX dynamics

**Figure 1:**
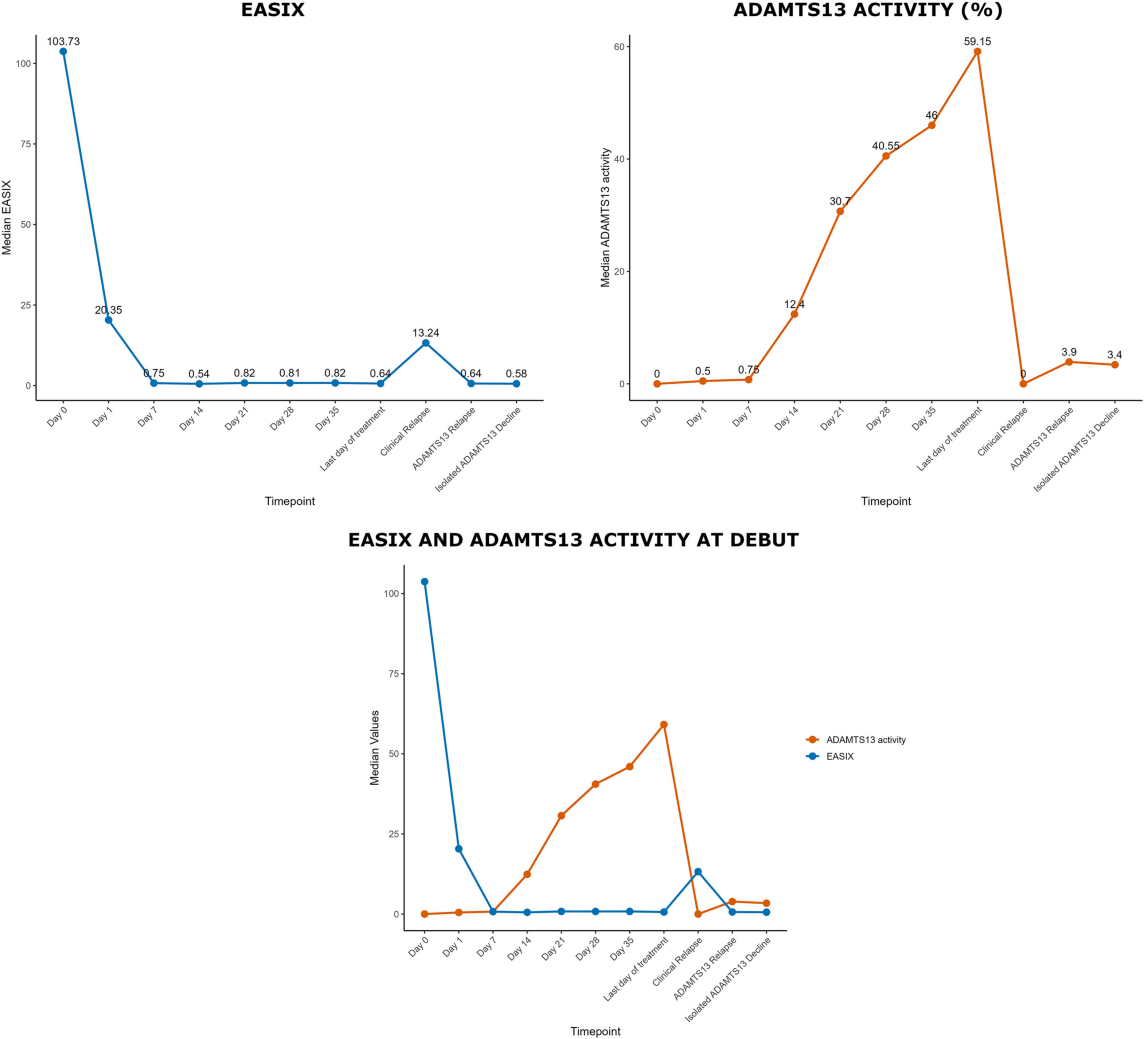
EASIX and ADAMTS13 activity dynamics at debut, clinical relapse, ADAMTS13 relapse, and isolated ADAMTS13 decline.

Laboratory findings for the entire cohort are summarized in **Table 2**. At diagnosis, median ADAMTS13 activity was 0% (IQR 0.0–0.7), and anti-ADAMTS13 antibody levels were 63 U/mL (IQR 38-83). A progressive increase in ADAMTS13 activity was observed from day 14, surpassing 20% by day 21 (**Figure 1**).

**Table 2:** Laboratory data (n=55).

Variable	Day 0	Day 1	Day 7	Day 14	Day 21	Day 28	Day 35	Last day of treatment	Clinical relapse	ADAMTS13 relapse	Isolated ADAMTS13 decline
Hemoglobin (g/L)	86.0 (71.5–99.5)	81.0 (71.0–91.0)	96.0 (86.0–101.0)	106.0 (97.2–113.7)	111.0 (103.5–122.5)	115.0 (102.7–127.5)	121.0 (109.0–128.0)	114.0 (103.0–127.0)	127.0 (90.0–142.0)	149.5 (136.0–154.7)	154.0 (137.0–156.0)
Hematocrit (%)	24.4 (21.0–28.3)	24.0 (20.7–27.0)	29.6 (27.1–30.6)	32.9 (29.8–35.1)	33.8 (30.8–37.4)	35.2 (32.2–38.7)	36.2 (34.3–38.5)	35.5 (31.9–39.0)	36.9 (31.5–42.1)	44.7 (40.5–45.5)	46.1 (41.8–46.2)
VCM (fL)	88.0 (85.0–92.7)	89.0 (86.7–93.2)	94.6 (91.6–98.4)	97.1 (93.6–100.3)	98.3 (93.6–101.5)	96.0 (93.3–99.3)	94.1 (89.0–98.7)	95.9 (94.0–100.0)	92.0 (90.9–95.2)	89.8 (89.4–92.5)	87.2 (84.5–90.2)
Reticulocytes (%)	5.6 (3.9–10.1)	8.0 (4.0–12.0)	9.2 (7.7–11.7)	4.2 (3.2–4.7)	3.2 (2.3–4.3)	2.0 (1.9–3.1)	2.7 (2.1–3.1)	2.8 (1.9–3.5)	3.5 (3.1–3.8)	1.3 (1.1–1.5)	1.7 (1.6–1.8)
Leukocytes (x10^9^/L)	10.3 (7.7–12.7)	11.7 (8.7–15.7)	12.4 (8.6–15.5)	12.7 (9.4–15.1)	10.6 (8.3–14.3)	9.7 (6.7–11.9)	9.4 (7.6–10.9)	9.1 (7.1–13.1)	10.5 (8.6–11.4)	7.1 (6.1–8.6)	8.4 (6.5–10.1)
Platelet counts (x10^9^/L)	13 (8–19)	29 (15–63)	248 (173–441)	271 (168–393)	203 (129–297)	209 (175–294)	253 (160–339)	257 (176–330)	42 (16–67)	228 (157–279)	281 (246–287)
Creatinine (mg/dL)	1.1 (0.8–1.4)	0.9 (0.7–1.2)	0.8 (0.7–1.0)	0.8 (0.7–0.9)	0.8 (0.7–0.8)	0.8 (0.7–0.9)	0.8 (0.6–0.8)	0.8 (0.6–0.9)	0.8 (0.7–1.0)	0.8 (0.6–1.0)	0.8 (0.6–0.9)
LDH (U/L)	1204 (818–2082)	528 (331–857)	228 (197–273)	196 (169–226)	196 (164–244)	216 (196–287)	208 (199–284)	211 (179–258)	521 (401–728)	164 (159–207)	189 (184–200)
EASIX	103.7 (41.1–290.9)	20.3 (8.3–61.3)	0.7 (0.4–1.0)	0.5 (0.3–1.1)	0.8 (0.4–1.2)	0.8 (0.6–1.2)	0.8 (0.4–1.5)	0.64 (0.4–1.1)	13.2 (5.0–27.5)	0.6 (0.5–0.9)	0.5 (0.5–0.6)
ADAMTS13 activity (%)	0.0 (0.0–0.7)	0.5 (0.0–2.2)	0.7 (0.0–10.3)	12.4 (2.0–27.9)	30.7 (0.9–60.1)	40.5 (15.9–51.3)	46.0 (22.0–62.1)	59.1 (27.0–71.3)	0.0 (0.0–0.8)	3.9 (1.0–8.2)	3.4 (0.0–6.0)

^1^Median (IQR)

LDH, lactate dehydrogenase.

At onset, the median EASIX was 103.7 (IQR 41.1-290.9). Following treatment initiation, EASIX values declined rapidly, reaching values below 1 by day 7 (**Figure 1**). Similarly, creatinine, LDH, and platelets counts normalized by day 7; however, substantial inter-individual variability was observed in these parameters compared to EASIX values (**Table 2** and **Supplementary Figure S1**).

During follow-up, clinical relapses, ADAMTS13 relapses, and isolated ADAMTS13 declines were all associated with ADAMTS13 activities below 10%. Notably, EASIX values remained consistently below one after initial recovery, except during clinical relapses, where a marked elevation was observed, with a peak median value of 13.2 (IQR 5.0-27.5).

### EASIX and refractoriness

**Figure 2:**
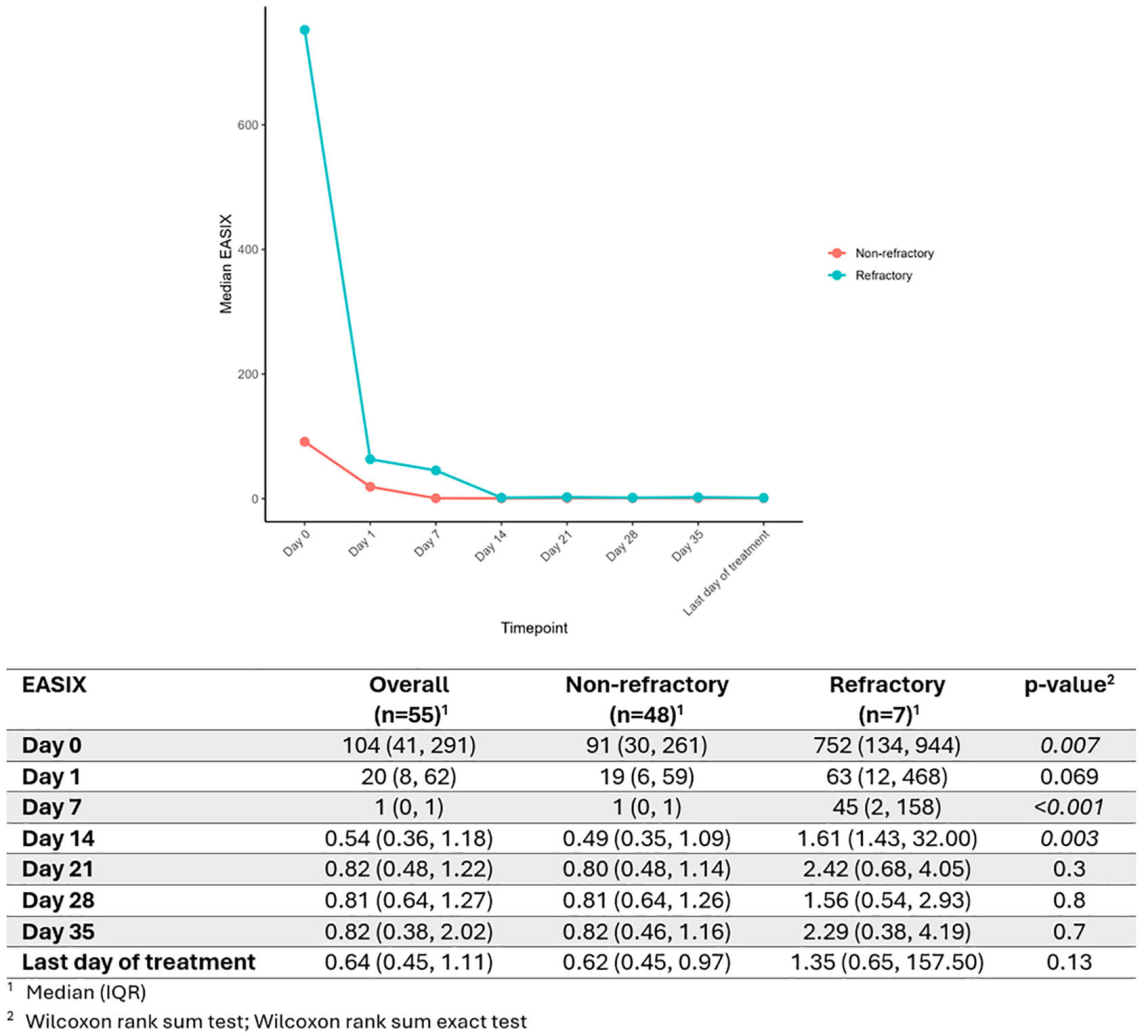
EASIX dynamics in non-refractory vs. refractory patients.

The study cohort was divided into two groups based on refractoriness to first-line treatment: non-refractory (n=48) and refractory (n=7; **Figure 2**). Refractory patients were slightly older (median age: 58 vs. 47 years), and included a lower proportion of females (57% vs. 63%). Four of the seven refractory patients presented neurological symptoms at diagnosis, while none exhibited gastrointestinal manifestations. Regarding treatment, 5/7 refractory patients received CS, TPE, rituximab, and caplacizumab; whereas the remaining two were treated with CS, TPE, and rituximab. At presentation, two patients developed infectious complications, one had cardiac complications, and two experienced hemorrhagic complications.

Median EASIX values were significantly higher in refractory patients at day 0 (91 vs. 752, p=0.007), day 7 (1 vs. 45, p<0.001), and day 14 (0.4 vs. 1.6, p=0.003). At day 1, a non-significant trend toward higher EASIX values was also observed in the refractory group (19 vs. 63, p=0.069).

Based on these findings, logistic regression models were used to evaluate the association between EASIX and refractoriness. Higher EASIX values at day 0 were significantly associated with an increased risk of refractoriness (β=0.003, SE = 0.001, *p* = 0.021), corresponding to an odds ratio (OR) of 1.003 (95% CI: 1.001–1.005). Similar analyses for subsequent time points revealed statistically significant associations for day 1 (OR = 1.009, 95% CI: 1.002–1.020, *p* = 0.041) and day 14 (OR = 45.6, 95% CI: 2.62–7765.2, *p* = 0.042), with a borderline result for day 7 (OR = 1.322, 95% CI: 1.042–1.860, *p* = 0.070).

**Figure 3:**
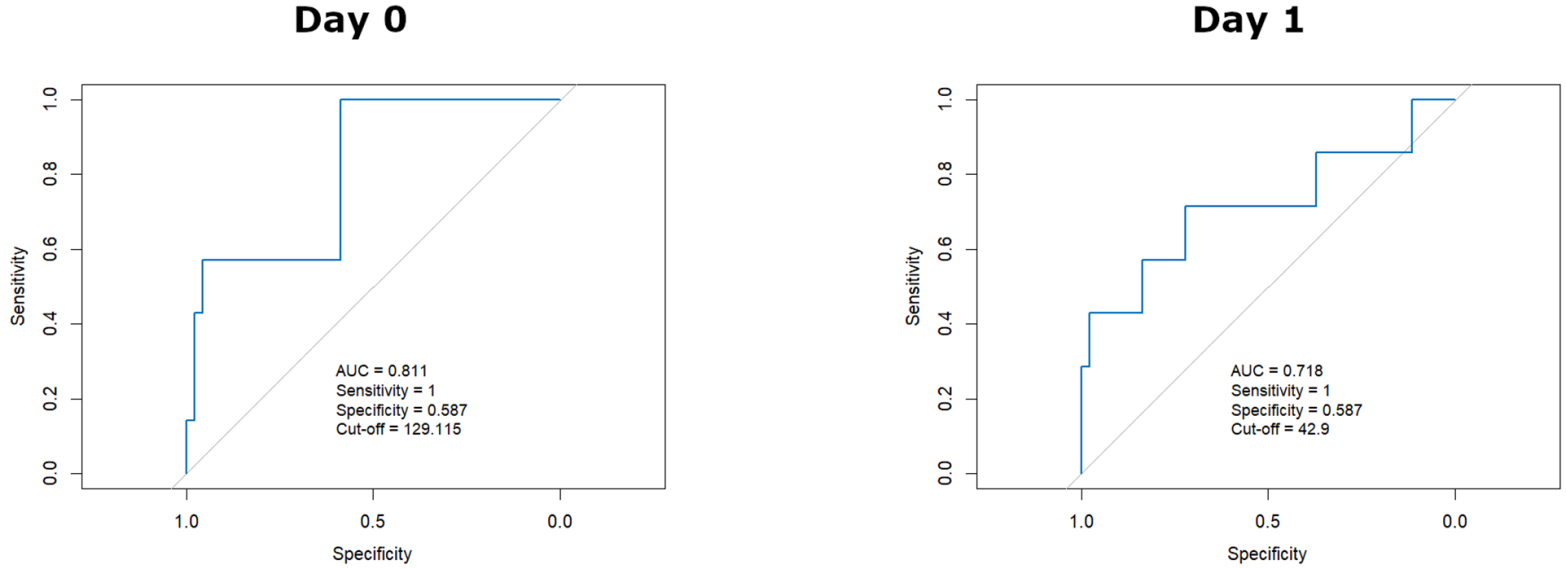
EASIX day 0 and day 1 ROC curves.

To explore the predictive performance of early EASIX values, ROC curve analyses were conducted focusing on days 0 and 1, given their clinical relevance for early risk stratification. The area under the ROC curve (AUC) was 0.811 at day 0, with an optimal cut-off value of 129.1 yielding 100% sensitivity (SS) and 58.7% specificity (SP). At day 1, the AUC was 0.718, with a SS of 71.4% and SP of 72.1% at a cut-off value of 42.9 (**Figure 3**).

To determine whether EASIX adds prognostic value beyond its individual components, logistic regressions were also conducted for platelet count and LDH at days 0 and 1 (**Supplementary Figure S2**). These parameters were analyzed separately as they are commonly used for clinical monitoring, unlike creatinine. Platelet counts showed no significant association with refractoriness (day 0: OR = 0.827, 95% CI: 0.627–0.974, p=0.097; day 1: OR = 0.987, 95% CI: 0.952–1.007, p=0.355). In contrast, higher LDH levels were significantly associated with refractoriness at both time points (day 0: OR = 1.001, 95% CI: 1.000–1.001, p=0.036; day 1: OR = 1.002, 95% CI: 1.000–1.003, p=0.017).

ROC analyses confirmed moderate predictive ability of LDH alone, with AUCs of 0.714 (day 0, SS 85.7% and SP 58.7%) and 0.764 (day 1, SS 71.4% and SP 71.4%), at thresholds markedly exceeding the normal reference values (1343 U/L and 1100 U/L, respectively; **Supplementary Figure S3**).

### EASIX and mortality

**Figure 4:**
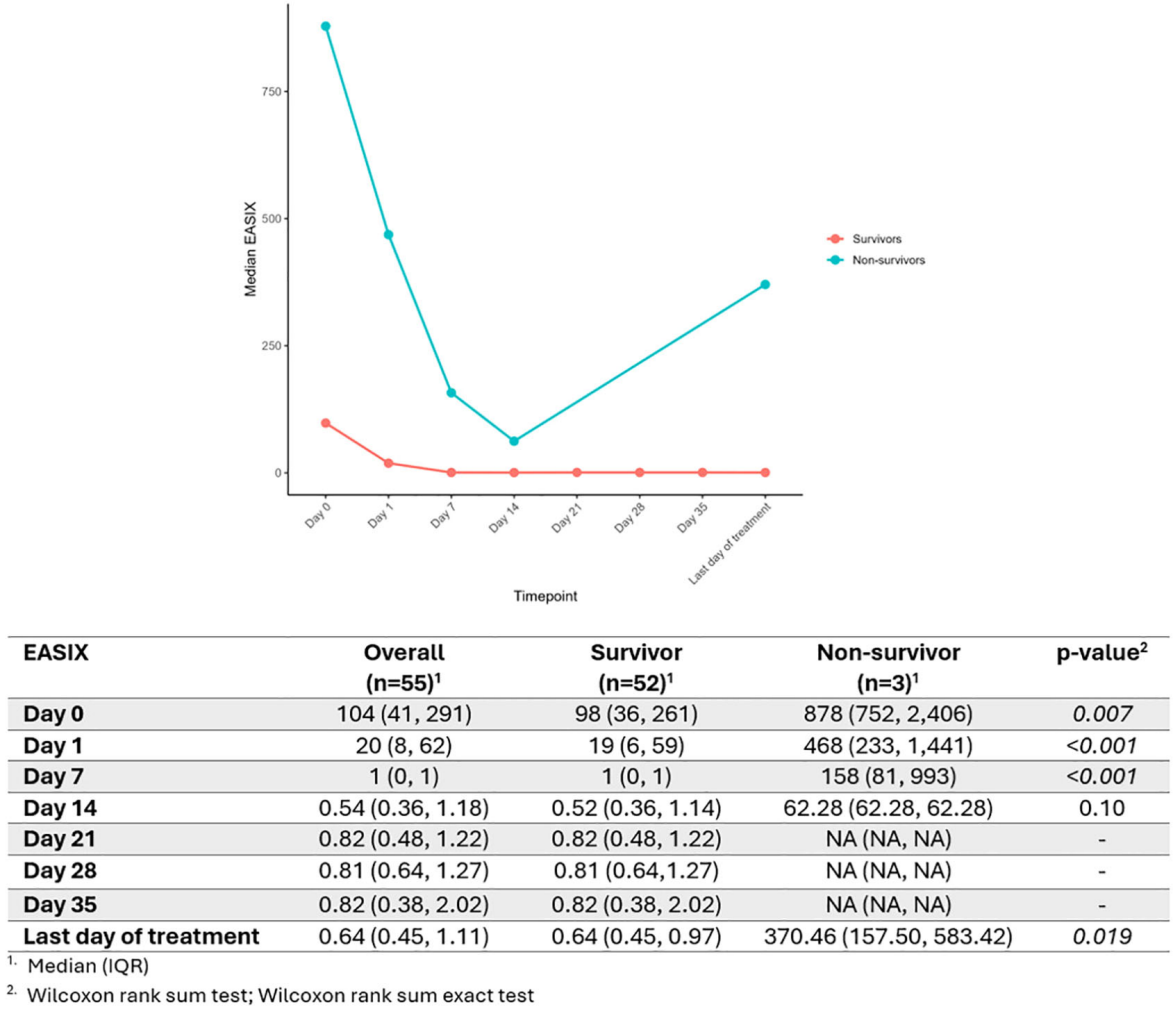
EASIX dynamics in survivors vs. non-survivors.

The cohort was divided based on mortality risk during the first year of diagnosis into survivors (n = 52) and non-survivors (n = 3). Among the deceased, causes of death included two cases related to refractoriness and one due to myocarditis. One patient who died after one year from diagnosis due to causes unrelated to iTTP was included in the 1-year survivors. Logistic regression analyses showed that EASIX measured at day 0 was significantly associated with mortality risk (β=0.004, SE = 0.002, p=0.027), with an OR of 1.004 (95% CI: 1.001–1.009). EASIX at day 1 showed a non-significant trend towards association with mortality (β=0.027, SE = 0.016, p=0.103; OR = 1.027, 95% CI: 1.008–1.099), **Figure 4**.

To evaluate whether the individual components of EASIX were also associated with mortality, additional logistic regression analyses were performed for platelet count and LDH at days 0 and 1. Platelet count showed no significant association with mortality (day 0: OR = 0.762, 95% CI: 0.432–0.990, p = 0.204; day 1: OR = 0.958, 95% CI: 0.842–1.007, p = 0.329). In contrast, higher LDH levels at day 0 were significantly associated with mortality risk (day 0: OR = 1.001, 95% CI: 1.000–1.002, p = 0.017; day 1: OR = 1.184, 95% CI: 0–10^17^, p = 0.998).

## Discussion

This study offers novel insights into the clinical utility of EASIX as an indirect biomarker of endothelial activation in the context of iTTP. Our analysis describes the evolving behavior of EASIX throughout the clinical course of iTTP and demonstrates its predictive capacity for refractoriness and mortality in this setting. Its early and pronounced response to therapy, along with its sharp increase during relapses, highlights its potential role as a real-time biomarker for risk stratification and therapeutic guidance.

The introduction of caplacizumab into the iTTP treatment landscape, owing to its ability to accelerate platelet recovery, has prompted a redefinition of response criteria and patient monitoring strategies (13). In this evolving context, ADAMTS13 activity has gained attention as a key biomarker for follow-up (2, 4, 5, 13). However, complete ADAMTS13 responses are not consistently achieved, and maintained low activity levels do not invariably lead to relapse (2, 29–31). Moreover, ADAMTS13 testing remains time-consuming, and is limited to specialized laboratories.

In this context, EASIX emerges as a promising biomarker. Originally developed to predict outcomes and endothelial complications after allogeneic stem cell transplantation, it has since been validated in multiple disease settings characterized by endothelial injury, such as multiple myeloma, lymphoma, myelodysplastic syndromes, COVID-19, and in patients treated with CAR-T cell therapies (14–27). These precedents reinforce the biological plausibility of its use in iTTP, a condition in which endothelial activation and microvascular thrombosis are central pathophysiological components. In contrast to ADAMTS13, EASIX relies exclusively on standard, widely available laboratory tests, offering a fast, inexpensive, and reproducible alternative with potential for broader clinical applicability. The cost-effectiveness and operational simplicity of EASIX make it particularly suitable for settings where access to ADAMTS13 testing is limited, delayed, or where rapid clinical decisions are required.

In our study, EASIX normalization occurred at day 7, preceding the restoration of ADAMTS13 activity (day 21), and showed less inter-patient variability than LDH and platelet count, a potential misleading variable in the era of caplacizumab, suggesting it may be a more reliable marker for early treatment monitoring.

Higher baseline EASIX values were strongly associated with refractoriness. Refractory patients exhibited significantly higher EASIX values at day 0 and maintained elevated values at days 1, 7, and 14, reflecting sustained endothelial stress. EASIX outperformed LDH and platelet count in predicting refractoriness. The platelet count showed no significant association, while LDH was significant but only moderately predictive, with AUCs of 0.714 and 0.764 at days 0 and 1, respectively. However, it required abnormally high thresholds, limiting its standalone clinical utility. In contrast, EASIX, by integrating multiple clinically relevant parameters, provides a more comprehensive assessment of endothelial dysfunction and global disease severity. ROC analyses of EASIX showed excellent discriminatory power at day 0 (AUC 0.81, 100% sensitivity), suggesting its potential role in early risk stratification and guiding therapeutic escalation.

Notably, EASIX also demonstrated value in relapse monitoring. It rose markedly in clinical relapses, but remained low in ADAMTS13 relapses and isolated ADAMTS13 declines, providing a helpful distinction between clinically relevant and subclinical events. Importantly, iTTP is a chronic disease with a high risk of relapse, which requires long-term, structured follow-up. Regular assessment of ADAMTS13 activity and EASIX should be considered part of post-remission surveillance, especially during situations that may trigger clinical relapse, such as infections, pregnancy, or systemic inflammatory episodes. Incorporating EASIX into routine follow-up could enhance early detection of endothelial stress and facilitate timely therapeutic intervention.

Beyond relapse monitoring, EASIX also showed a potential association with mortality. In our cohort, higher baseline EASIX values at day 0 were significantly linked to an increased risk of death, and LDH at day 0 demonstrated a similar trend, suggesting that both may reflect severe endothelial injury and global disease burden. However, since most deaths in our series were related to refractoriness, these findings should be interpreted with caution and warrant confirmation in larger, independent studies.

Nevertheless, this study has limitations that should be recognized, including its retrospective design and small sample size. In addition, the heterogeneity observed in treatment regimens and ADAMTS13 assay methodology across centers may introduce variability among results.

In conclusion, EASIX emerges as a sensitive and accessible biomarker of endothelial injury in iTTP, outperforming traditional markers. By integrating LDH, creatinine, and platelet count over time, EASIX allows dynamic monitoring of disease activity, evaluation of treatment response, and, most importantly, early detection of clinical relapses. Moreover, its capacity to predict refractoriness, and, potentially, mortality, further underscores its value for risk stratification, and treatment guidance, including decisions on therapy intensification or early implementation of targeted approaches. Although prospective validation is warranted, these findings support the integration of EASIX into future risk-adapted therapeutic algorithms for iTTP.

## Data Availability

The original contributions presented in the study are included in the article/**Supplementary Material**. Further inquiries can be directed to the corresponding author.
