# Supplementary material for: EASIX, a new tool to predict response and refractoriness in immune-mediated thrombotic thrombocytopenic purpura
Source: Front Immunol. 2025 Nov 17;16:1700907. doi: 10.3389/fimmu.2025.1700907 (PMC12665919; doi:10.3389/fimmu.2025.1700907)

SUPPLEMENTARY MATERIAL

FIGURE S1. Creatinine, LDH and platelet dynamics at debut, clinical relapse, ADAMTS13 relapse and isolated ADAMTS13 decline

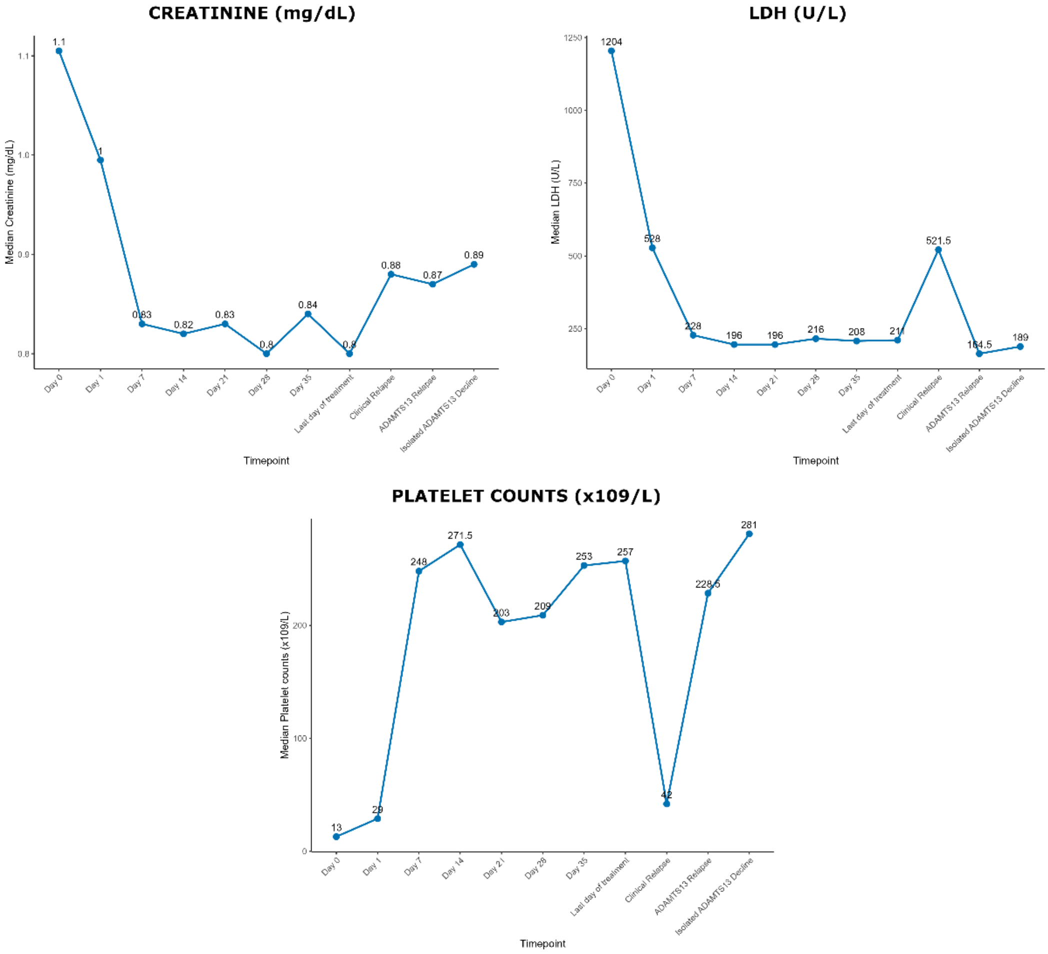

FIGURE S2. Platelets and LDH dynamics in refractory vs. non-refractory

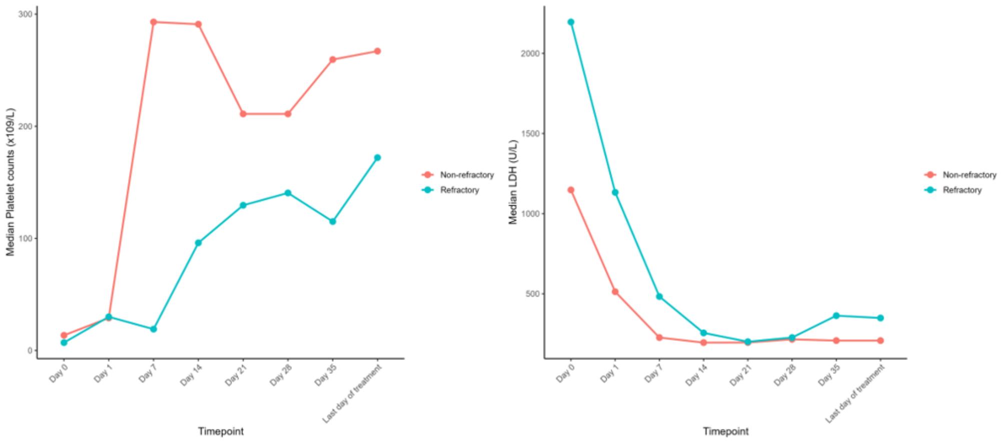

Platelet counts ($x10^9$/L)	Overall (n=55)[1]	Non-refractory (n=48)[1]	Refractory (n=7)[1]	p-value[2]
**Day 0**	13 (8, 20)	13 (8, 22)	7 (7, 8)	0.088
**Day 1**	29 (15, 63)	29 (15, 67)	16 (8, 34)	0.2

LDH (U/L)	Overall (n=55)[1]	Non-refractory (n=48)[1]	Refractory (n=7)[1]	p-value[2]
**Day 0**	1,204 (818, 2,082)	1,187 (807, 2,067)	4,123 (2,196, 5,215)	0.015
**Day 1**	528 (329, 862)	513 (329, 842)	2,696 (2,196, 8,060)	0.004

1. Median (IQR)
2. Wilcoxon rank sum test; Wilcoxon rank sum exact test

FIGURE S3. LDH day 0 and day 1 ROC curves

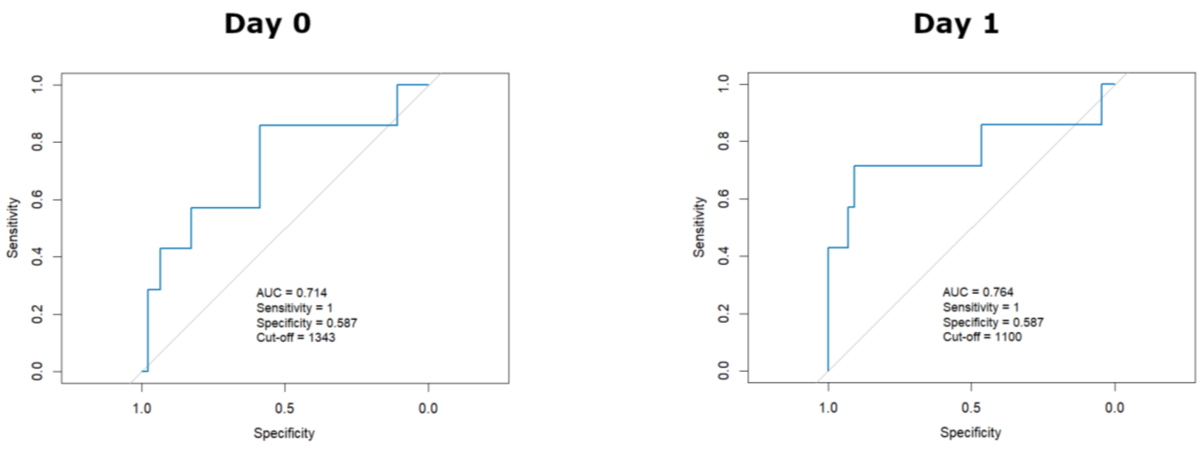

Supplement: Supplementary file 1 [file SupplementaryFile1.pdf]
